# Effects of huperzine A on hippocampal inflammatory response and neurotrophic factors in aged rats after anesthesia ^1^

**DOI:** 10.1590/s0102-865020190120000005

**Published:** 2020-02-07

**Authors:** Yi Cai, Penghan Huang, Yizu Xie

**Affiliations:** I Master, Department of Anesthesiology, Yongchuan Hospital, Chongqing Medical University, Chongqing 402160, China. Design of the study, statistics analysis, final approval.; II Bachelor, Department of Anesthesiology, People’s Hospital of Bishan District, Chongqing 402760, China. Acquisition of data, final approval.; III Bachelor, Department of Anesthesiology, People’s Hospital of Bishan District, Chongqing 402760, China. Design of the study, critical revision, final approval.

**Keywords:** Isoflurane, Cognition, Anesthesia, Rats

## Abstract

**Purpose:**

To investigate the effects of huperzine A (HupA) on hippocampal inflammatory response and neurotrophic factors in aged rats after anesthesia.

**Methods:**

Thirty-six Sprague Dawley rats (20-22 months old) were randomly divided into control, isofluran, and isoflurane+HupA groups; 12 rats in each group. The isoflurane+HupA group was intraperitoneally injected with 0.2 mg/kg of HupA. After 30 min, isoflurane inhalation anesthesia was performed in the isoflurane and isoflurane+HupA groups. After 24 h from anesthesia, Morris water maze experiment and open-field test were performed. Hippocampal inflammatory and neurotrophic factors were determined.

**Results:**

Compared with isoflurane group, in isofluran+HupA group the escape latency of rats was significantly decreased (P < 0.05), the original platform quadrant residence time and traversing times were significantly increased (P < 0.05), the central area residence time was significantly increased (P < 0.05), the hippocampal tumor necrosis factor α, interleukin 6 and interleukin 1β levels were significantly decreased (P < 0.05), and the hippocampal nerve growth factor, brain derived neurotrophic factor and neurotrophin-3 levels were significantly increased (P < 0.05).

**Conclusion:**

HupA may alleviate the cognitive impairment in rats after isoflurane anesthesia by decreasing inflammatory factors and increasing hippocampal neurotrophic factors in hippocampus tissue.

## Introduction

Postoperative cognitive dysfunction (POCD) is commonly seen in elderly patients after surgery. It refers to the neurodegenerative changes caused or aggravated by anesthesia, surgery and other factors. The main clinical manifestations of POCD are decreased orientation, learning and memory abilities or disturbance of consciousness^1^ . POCD can cause delayed rehabilitation, increased complications, and even loss of self-care ability, which prolongs the hospitalization duration and increases the medical costs^2^ . This disease can last for several weeks, several years or even the whole life. With the progress of medical conditions and levels, the number of elderly patients receiving surgical treatment is increasing, with the increased incidence of POCD^3^ . Isoflurane is a commonly used inhalation anesthetic in the clinic. It has outstanding analgesic effects and good controllability^4^ . However, current study has shown that the isoflurane inhalation anesthesia can increase the incidence of POCD in elderly patients, and its mechanism is still unclear^5^ . Huperzine A (HupA) is an alkaloid extracted from the Chinese endemic plant *Serrate Clubmoss* herb. It has the advantages of low molecular weight and high fat solubility, and easily penetrates the blood-brain barrier. After entering the central nervous system, HupA is mainly distributed in the frontal lobe and temporal lobe of the brain, with multi-target pharmacological effect^6^ . Animal experiments have shown that HupA can improve the learning and memory abilities, enhance the cholinesteryl acetyltransferase activity, and increase the antioxidant activity of neurons^7 - 8^ . In the clinic, HupA is used to alleviate the amnesia symptoms in patients with Alzheimer’s disease^9^ . This study was designed to investigate the effect of Huperzine A on cognitive function of aged rats after anesthesia and the related mechanisms.

## Methods

This study was performed with the approval of ethics committee of Chongqing Medical University. All animal procedures followed the Guide for the Care and Use of Laboratory Animals by the National Institutes of Health.

Thirty-six SPF-grade Sprague-Dawley rats (20-22 months old; 500-600 g; male) were randomly divided into control, isoflurane, and isoflurane+HupA groups, with 12 rats in each group. The rats in isoflurane+HupA group were intraperitoneally injected with 0.2 mg/kg of HupA (Henan Zhulin Zhongsheng Pharmaceutical Co., Ltd., Zhengzhou, China). The control and isoflurane groups were given by intraperitoneal injection an equal volume of normal saline. After 30 min, the rats in three groups were placed in the anesthesia box, respectively. The intake of anesthesia box was connected with the anesthesia machine to introduce the isoflurane, and the outlet of anesthesia box was connected with a multi-functional anesthesia detector to identify the concentration of isoflurane. The rats in isoflurane and isoflurane+HupA groups inhaled 2.5% isoflurane (using air-oxygen mixture containing 60% oxygen as carrier) for 3 min, followed by inhalation of 1.5% isoflurane for 4 h. The rats in control group only inhaled air-oxygen mixture containing 60% oxygen for 4 h.

### Morris water maze experiment

After 24 h from anesthesia, the Morris water maze experiment was performed in all rats in the morning^10^ . An elliptical pool (diameter 120 cm, height 60 cm) was used as the Morris water maze. The water was opaque. The water temperature was 22-26^o^C. The positioning navigation experiment was conducted for 4 days (day 1, 2, 3 and 4). On each day, the rats were put into water, facing the wall, from different quadrants. The time from rats entering the water to climbing on the platform was recorded. The time limit was 60s, and the time of rats that could not find the platform within 60s was recorded as 60s. The average time of rats entering the water from four quadrants was recorded as the escape latency. On day 5, the platform was removed, and the spatial probe test was conducted. The rats were put into the water from the third quadrant (any quadrant, the same for all animals). The time of rats exploring the original platform quadrant within 60s (original platform quadrant exploring time) and the times of rats traversing the original platform quadrant within 60s (original platform quadrant traversing times) were recorded.

### Open-field test

Open-field test was conducted in the afternoon of each day performing Morris water maze experiment according to the reported method^11^ . At the beginning of the experiment, the rats were placed in the center of the open-field box. The rats were allowed to act freely. The duration of rats in the central area within 15 min was recorded as the central area residence time. Between each rat, the open-field box was thoroughly cleaned to avoid the interference.

### Determination of hippocampal inflammatory and neurotrophic factors

After the behavioral test, the rats were executed by cervical dislocation. The head was cut off and the hippocampus was taken completely. After weighing, the hippocampus tissue was homogenized at 4^o^C, followed by centrifuging at 2000 r/min for 15 min. The supernatant was taken and stored. The levels of inflammatory factors including tumor necrosis factor α (TNF-α), interleukin 6 (IL-6) and interleukin 1β (IL-1β) and neurotrophic factors including nerve growth factor (NGF), brain derived neurotrophic factor (BDNF) and neurotrophin-3 (NT-3) were determined by enzyme-linked immunosorbent assay. The processes were according to the instructions in the kits (Fuzhou Maixin Biotechnology Development Co., Ltd., Fuzhou, China).

### Statistical analysis

All statistical analysis was carried out using SPSS 18.0 software (SPSS Inc., Chicago, IL, USA). Results were expressed as mean±standard deviation. Comparisons among three groups were carried out by analysis of variance (ANOVA) with LSD-t test used for post-hoc analysis. P < 0.05 was deemed to be statistically significant.

## Results

### Effects of HupA on escape latency of rats after anesthesia

**Figure 1:**
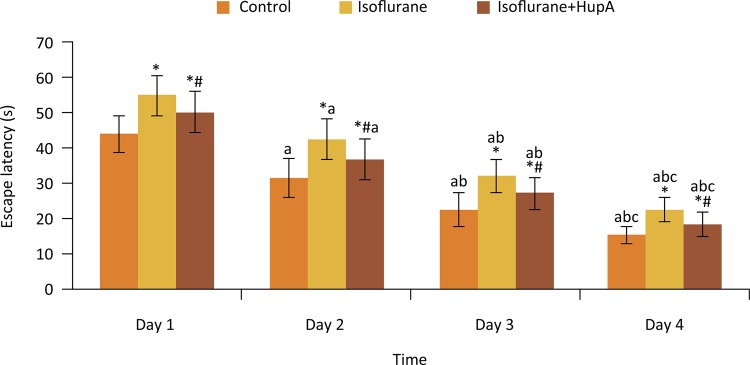
Effects of HupA on escape latency of rats after anesthesia (s). *P < 0.05 compared with control group; #P < 0.05 compared with isoflurane group; aP < 0.05 compared with day 1; bP < 0.05 compared with day 2; cP < 0.05 compared with day 3.

Effects of HupA on escape latency in rats after anesthesia were shown in Figure 1 . From day 1 to day 4, the escape latency of rats in each group was gradually decreased, with significant difference among four time points (P < 0.05). At each time point, the escape latency of rats in isoflurane and isoflurane+HupA groups were significantly higher than that in control group (P < 0.05). Compared with isoflurane group, the escape latency in isoflurane+HupA group at each time point was significantly decreased (P < 0.05).

### Effects of HupA on original platform quadrant exploring time and traversing times of rats after anesthesia

**Figure 2:**
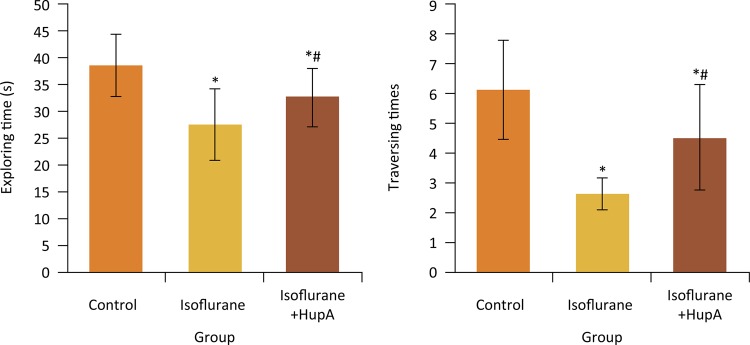
Effects of HupA on original platform quadrant exploring time and traversing times of rats after anesthesia. *P < 0.05 compared with control group; #P < 0.05 compared with isoflurane group.

As shown in Figure 2 , compared with the control group, the original platform quadrant exploring time and traversing times of rats in isoflurane and isoflurane+HupA groups were significantly decreased, respectively (P < 0.05). Compared with isoflurane group, the original platform quadrant exploring time and traversing times in isoflurane+HupA group were significantly increased, respectively (P < 0.05).

### Effects of HupA on central area residence time of rats after anesthesia

**Figure 3:**
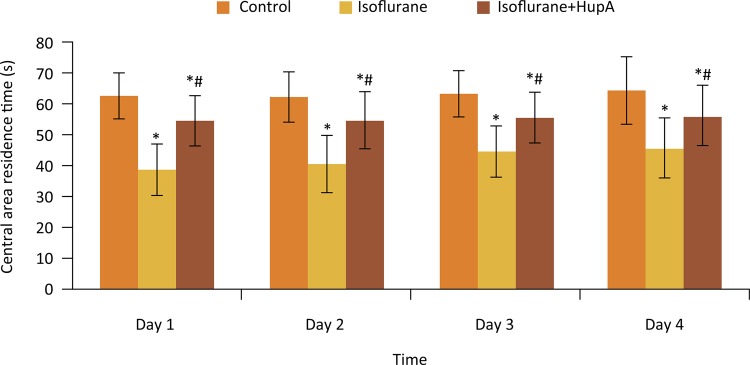
Effects of HupA on central area residence time of rats after anesthesia (s). *P < 0.05 compared with control group; #P < 0.05 compared with isoflurane group.

The central area residence time of rats in each group did not obviously change from day 1 to day 4. At each time point, the central area residence time of rats in isoflurane and isoflurane+HupA groups were significantly lower than that in control group (P < 0.05). Compared with isoflurane group, the central area residence time in isoflurane+HupA group at each time point was significantly increased (P < 0.05) ( Fig. 3 ).

### Effects of HupA on hippocampal TNF-α, IL-6 and IL-1β levels of rats after anesthesia

**Figure 4:**
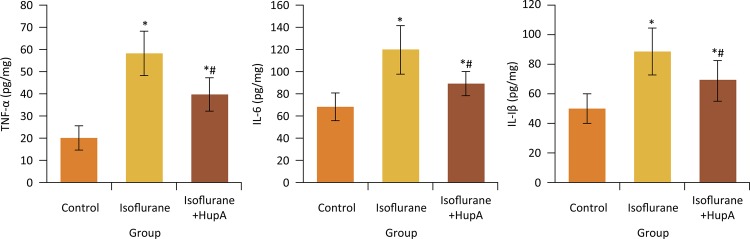
Effects of HupA on hippocampal TNF-α, IL-6 and IL-1β levels of rats after anesthesia. *P < 0.05 compared with control group; #P < 0.05 compared with isoflurane group. TNF-α, tumor necrosis factor α; IL-6, interleukin 6; IL-1β, interleukin 1β.

Figure 4 showed that, compared with control group, the hippocampal TNF-α, IL-6 and IL-1β levels in isoflurane and isoflurane+HupA groups were significantly increased, respectively (P < 0.05). Compared with isoflurane group, the TNF-α, IL-6 and IL-1β levels in isoflurane+HupA group were significantly decreased, respectively (P < 0.05).

### Effects of HupA on hippocampal NGF, BDNF and NT-3 levels of rats after anesthesia

**Figure 5:**
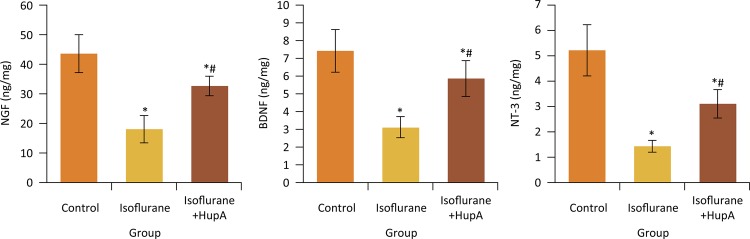
Effects of HupA on hippocampal NGF, BDNF and NT-3 levels of rats after anesthesia. *P < 0.05 compared with control group; #P < 0.05 compared with isoflurane group. NGF, nerve growth factor; BDNF, brain derived neurotrophic factor; NT-3, neurotrophin-3.

Compared with control group, the hippocampal NGF, BDNF and NT-3 levels in isoflurane and isoflurane+HupA groups were significantly decreased, respectively (P < 0.05). Compared with isoflurane group, the NGF, BDNF and NT-3 levels in isoflurane+HupA group were significantly increased, respectively (P < 0.05) ( Fig. 5 ).

## Discussion

POCD is a common complication of central nervous system after anesthesia, which is more likely to occur in elderly patients. Learning and memory functions are two important aspects of cognition ability^12^ . Morris water maze is a model for studying the learning and memory functions related to hippocampus, of which the positioning navigation test and spatial probe test are performed^13^ . Open-field test, also known as open-box experiment, is used to evaluate the autonomous behavior, exploratory behavior and tension of animals in new environments^14^ . These methods are commonly used to study the cognitive impairment caused by anesthesia or surgery^15 , 16^ . Previous studies have shown that HupA can ameliorate the impaired memory and improve the cognitive functions of animals^17 , 18^ . This has given us a great inspiration. Many labs use isoflurane in aged rats and mice to examine cognitive function^19 , 20^ . In our study, the effects of HupA on cognitive function in aged rats after anesthesia with isoflurane were investigated. Results indicate that, the isoflurane anesthesia can cause the cognitive impairment of rats, and the HupA pre-treatment can alleviate the cognitive impairment of rats after anesthesia.

At present, the mechanism of POCD occurrence and development is not completely clear. Studies have confirmed that the mechanism of cognitive impairment induced by isoflurane anesthesia in aged animals is related to the excessive release of hippocampal inflammatory factors^21 , 22^ . TNF-α, IL-6 and IL-1β are the common inflammatory factors. TNF-α has the neurodegenerative effect. It can inhibit the long-term potentiation in hippocampus and continuously increase the synaptic efficiency between neurons, thus reducing the cognitive function^23^ . IL-6 can affect the cognitive function through acting with the synaptic plasticity^23^ . IL-1β can activate caspase-3 and induce the neuronal apoptosis^23^ . Handattu *et al* .^24^ have found that TNF-α and IL-1β can induce the inflammatory response in hippocampus, which leads to the decreased cognitive function. Reducing their levels can significantly improve the cognitive function. Trapero and Cauli^25^ have also found that the change of serum IL-6 level has a correlation with the occurrence of cognitive dysfunction. Results of our study indicate that the inflammatory reaction in hippocampus is involved in cognitive impairment of rats after isoflurane anesthesia. HupA can alleviate the cognitive impairment by decreasing the inflammatory reaction.

Neurotrophic factors are a class of small molecular polypeptides that play special roles in the development of the central nervous system. They can promote the proliferation, growth, differentiation and survival of nerve cells, and regulate the plasticity of synapses^26^ . NGF, BDNF and NT-3 are the most important neurotrophic factors. NGF can activate the cell metabolism by binding to specific TrkA receptor, promoting the proliferation and differentiation of nerve cells, and regulating the survival of central and peripheral nerve cells and the growth of axons, thus playing an important role in the repair of nerve cell injury^27^ . BDNF plays a neurotrophic role by binding to its specific high-affinity TrkB receptor^28^ . NT-3 can support the survival of sensory motor neurons, promote the development of neural crest cells, and regulate the occurrence and number of peripheral sensory neurons^29^ . NGF, BDNF and NT-3 can improve the learning and memory abilities and promote the growth of neurons in rat model of Alzheimer’s disease^30^ . Results of our study suggest that HupA can increase the hippocampal NGF, BDNF and NT-3 levels, which is related to its protective effect on cognitive impairment of rats after isoflurane anesthesia.

## Conclusions

HupA can alleviate the cognitive impairment after isoflurane anesthesia in rats, which may be related to its decreasing hippocampal inflammatory factors and increasing hippocampal neurotrophic factors in hippocampus tissue. This study has provided an experimental basis for clinical application of HupA to prevention of cognitive impairment after anesthesia. In this study, the HupA dose and administration time are based on results of our pre-experiments, which have differences with those in a previous study^31^ . In the next, these parameters should be further optimized. In addition, in the clinic, more aged females suffer from Alzheimer’s disease^32^ . In this study we only used the aged male rat in the experiment. The effects of HupA on cognitive function of aged female rats after anesthesia should be investigated in further studies. The correlations between biomarkers and performance levels have not been investigated. This should be considered in next studies.

## Footnotes

1 Research performed at Laboratory, Yongchuan Hospital, Chongqing Medical University, Chongqing 402160, China.
